# Association between neuromuscular electrical stimulation and intermittent pneumatic compression for the prevention of deep venous thrombosis among neurosurgery patients

**DOI:** 10.1080/07853890.2026.2752675

**Published:** 2026-10-29

**Authors:** Ling-Ling Wang, Li Ye, Li-Juan Rong, Wei-Yang Qian, Xiao-Li Chen, Zi-Rong Cheng, Shi-Yi Chen, Yu-pei Yang, Ching-Wen Chien, Tao-Hsin Tung

**Affiliations:** a Department of Neurosurgery, Taizhou Hospital of Zhejiang Province Affiliated to Wenzhou Medical University, Taizhou, China; b Institute of Educational Sciences, Hunan University, Changsha, China; c School of Journalism and Communication, Hunan University, Changsha, China; d Evidence-Based Medicine Center, Taizhou Hospital of Zhejiang Province Affiliated to Wenzhou Medical University, Taizhou, China; e Department of Hematology, Taizhou Hospital of Zhejiang Province, Wenzhou Medical University, Linhai, China; f Institute for Hospital Management, Tsinghua University, Shenzhen, China

**Keywords:** Deep vein thrombosis, neuromuscular electrical stimulation, intermittent pneumatic compression

## Abstract

**Background:**

Deep vein thrombosis (DVT) considerably affects the incidence and mortality rates in patients undergoing neurosurgery. Neuromuscular electrical stimulation (NMES) and intermittent pneumatic compression (IPC) are frequently used as adjunct treatments to prevent DVT; however, their effects remain unclear. This study was conducted to compare the association of NMES and IPC as two mechanical prophylaxis strategies in neurosurgical patients who were ineligible for pharmacological prophylaxis.

**Methods:**

Postoperative neurosurgical patients were retrospectively enrolled from a single centre between January 2020 and December 2021. These patients were assessed to have a high bleeding risk and received either NMES or IPC as mechanical prophylaxis. Univariable analysis was used to explore the risk factors for DVT, and multivariable logistic regression analysis was used to determine the independent associations between DVT and potential variables after adjusting for confounding factors.

**Results:**

Among 232 patients who underwent neurosurgery, 131 received IPC and 101 received NMES, with no overlap between the two groups. DVT was diagnosed in 62 patients (11 received NMES and 51 received IPC). After controlling for sex, age, operation duration and length of hospital stay, NMES use was associated with a lower incidence of DVT (OR = 0.268, 95% CI = 0.125–0.576, *p* = .001). Moreover, men were less likely to have DVT than women (OR = 0.469, 95% CI = 0.242–0.910, *p* = .025), older age was associated with higher odds of DVT (OR = 3.419, 95% CI = 1.594–7.334, *p* = .002), and a longer operation duration was associated with higher odds of DVT (OR = 2.180, 95% CI = 1.042–4.560, *p* = .039).

**Conclusion:**

This study found an association between NMES and reduced odds of DVT compared with IPC in neurosurgery patients. Although NMES may be more widely adopted in neurosurgery to decrease the incidence of DVT, further evidence is required to determine its effectiveness in clinical practice.

## 1. Introduction

Venous thromboembolism (VTE) is the third most common cause of vascular mortality worldwide and includes deep vein thrombosis (DVT) and pulmonary embolism (PE) [1]. The incidence of VTE in Western countries is approximately 0.87–1.82 per 1000 person-years [2]. In clinical practice, DVT is more than three times more common than PE; approximately two-thirds of patients with VTE present with DVT, whereas only one-third present with PE [3]. DVT and PE are the two most common complications of neurosurgery, and both may be the primary causes of death [4].

DVT can progress to PE, which is associated with high mortality rates in neurosurgery, but it can be significantly reduced by preventive measures [5]. Compared with patients undergoing spinal surgery, those undergoing intracranial surgery have a significantly greater risk of VTE [6]. The implementation of antithrombotic prophylaxis measures in neurosurgery can significantly reduce the incidence of VTE from 30% to approximately 1.5%–6% [6]. Therefore, prevention of DVT has become one of the most important clinical objectives of neurosurgery. Anticoagulants and mechanical prophylaxis are the main techniques used to prevent DVT and their combination is effective in high-risk patients [7]. In neurosurgery, anticoagulant therapy increases the risk of intracranial haemorrhagic complications [8]. Consequently, mechanical prophylaxis has emerged as the primary strategy for preventing DVT in patients undergoing neurosurgery.

Mechanical prophylaxis for DVT includes graduated compression stockings (GCS), intermittent pneumatic compression (IPC) and neuromuscular electrical stimulation (NMES) systems [9]. The first two have become the main strategies for preventing DVT in neurosurgical patients [10]. IPC is a method of increasing lower-limb blood circulation, thereby reducing the risk of VTE, by applying pressure to the legs and veins using inflatable cuffs in a commercial device [11]. GCS are medical-grade compression stockings designed to provide graduated pressure from the ankle to the thigh to promote blood circulation in the lower limbs [12]. External stress exerted by the GCS and IPC makes it difficult to act on deep veins, resulting in poor effectiveness of DVT prophylaxis. This external stress can also easily cause discomfort, such as fever and common peroneal nerve paralysis, leading to poor patient compliance [13]. To compensate for the lack of traditional mechanical prevention techniques, researchers have developed NMES. The principle that helps avoid DVT is to stimulate the common peroneal nerve of the lower limbs through the skin to cause deep leg muscle contractions, which significantly increase venous blood flow [14]. NMES may offer a beneficial therapeutic option for patients in whom pharmacological or conventional mechanical prophylaxis methods cannot be used because of contraindications, safety concerns, or impracticality [15]. Although several studies have demonstrated that NMES can decrease venous stasis, there is an ongoing debate regarding its clinical efficacy and effectiveness in preventing DVT [9].

Concerns regarding complications that may lead to bleeding limit the selection and implementation of preventive measures in neurosurgical patients [16]. Both NMES and IPC are physical prophylaxis agents without the risk of bleeding and are used in clinical practice to prevent DVT [17,18]. Furthermore, research has shown that NMES increases leg venous flow and velocity more effectively than IPC in healthy patients [19], but more clinical studies are required. In this hospital-based study, the thromboprophylactic associations of NMES and IPC were compared in patients undergoing neurosurgery in Linhai, China.

Published haemodynamic studies suggest a greater augmentation of lower-limb venous outflow with NMES than with IPC. However, direct comparative clinical data between these two mechanical prophylaxis modalities remain limited, specifically for neurosurgical patients at high bleeding risk for whom pharmacological thromboprophylaxis is not feasible. Accordingly, the a priori hypothesis was that NMES, compared with IPC, would be associated with a reduced risk of postoperative DVT in this patient population. This retrospective single-center study was therefore undertaken to explore this hypothesis.

## 2. Materials and methods

### 2.1. Study population

This retrospective study was conducted at the Department of Neurosurgery, Taizhou Hospital, Zhejiang Province, China. Data were collected from patients admitted between January 1, 2020 and December 31, 2021. Data collection began after ethical approval was granted on November 30, 2022 and was completed by the end of 2023. Data analysis and manuscript writing were conducted between 2023 and 2024.

A total of 4093 patients admitted to the Department of Neurosurgery at Taizhou Hospital between January 2020 and December 2021 were retrospectively reviewed. Adult inpatients who received either NMES or IPC as mechanical prophylaxis, underwent two sessions of lower-limb venous B-scans during hospitalization and had complete clinical data available were considered for inclusion. Patients were excluded if they had pre-existing lower-limb DVT upon admission, had physician-assessed contraindications to NMES or IPC (including patient refusal of mechanical prophylaxis), received any prophylactic anticoagulation during hospitalization, failed to complete two-session lower-limb venous B-scan examinations, or had non-diagnostic ultrasound images or incomplete key clinical records. After these exclusions, 232 patients were included in the final analysis (Figure 1). For all the patients included in this analysis, NMES or IPC was applied as the sole mechanical prophylactic modality. None of the patients received graduated compression stockings (GCS) during the study period as these were not part of the institutional standard protocol for neurosurgical patients. Both groups received identical routine nursing care, including early mobilization when clinically feasible, according to standardized postoperative rehabilitation protocols. This study was approved by the Ethics Committee of Taizhou Hospital, Zhejiang Province (approval number: K20221116; approval date: 30 November 2022 and approval number: K20260944; approval date: 18 September 2026). The study was conducted in accordance with the principles of the Declaration of Helsinki. Owing to the retrospective nature of the study and the use of de-identified patient data, the Ethics Committee of Taizhou Hospital, Zhejiang Province granted a waiver of informed consent, as documented in the approved application submitted to the committee. Patient confidentiality was strictly maintained and all data were anonymized prior to analysis. This study was registered in the Chinese Clinical Trial Registry (ChiCTR; https://www.chictr.org.cn/; registration number: ChiCTR2600132946).

**Figure 1.**
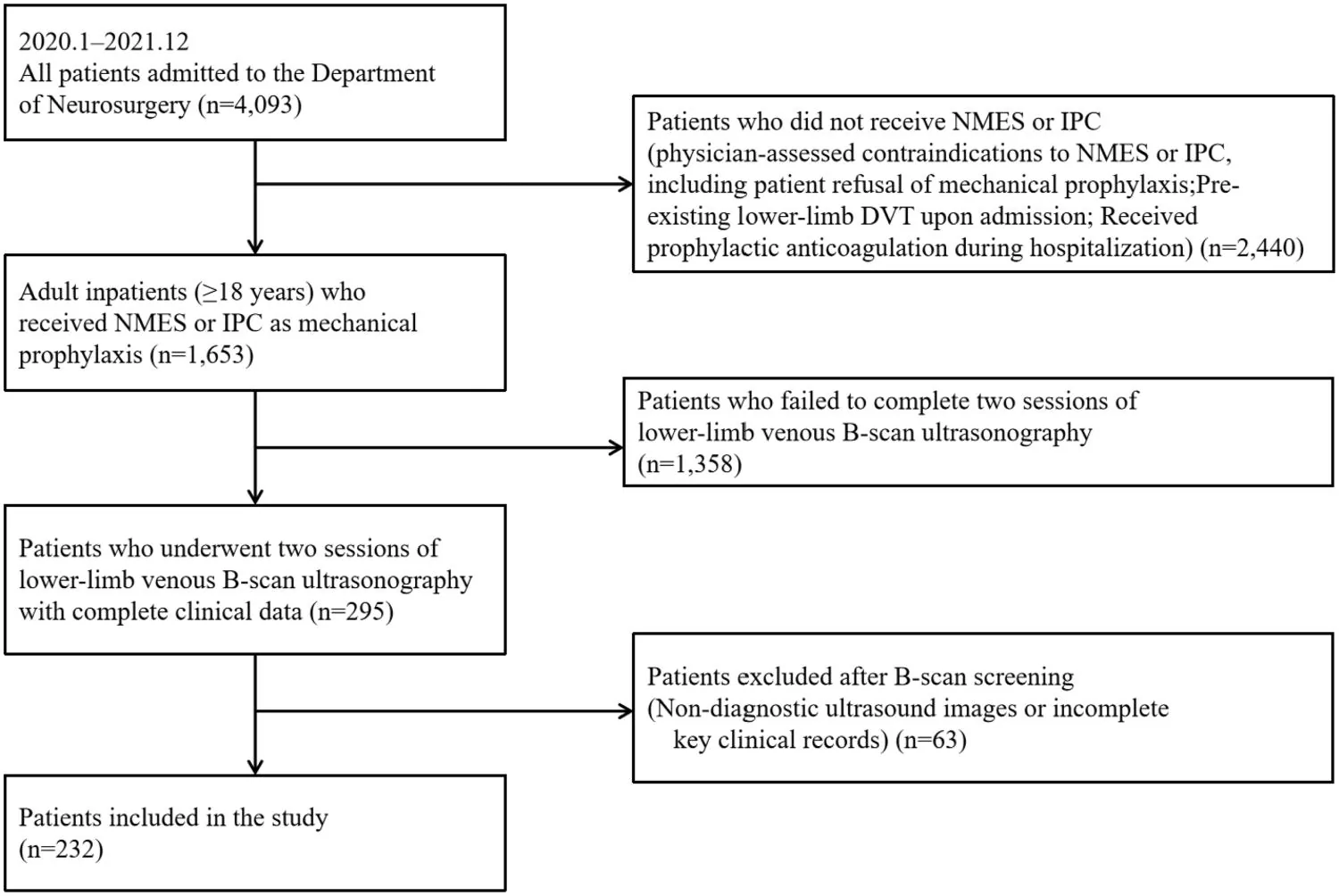
The process of data collection (from 4093 initial neurosurgical admissions, patients were excluded if they did not receive NMES or IPC, received prophylactic anticoagulation, did not undergo two routine lower‑limb ultrasound examinations, or had unavailable ultrasound data. After these exclusions, 232 patients were included in the final comparative analysis).

### 2.2. Data collection

The following data were retrospectively collected from the patients’ electronic medical records: age, sex, length of hospital stay, number of underlying diseases, operation duration, VTE risk score and DVT prophylaxis intervention. There were no missing data for any of the variables included in the final analysis, as all data were extracted from structured electronic medical records.

The VTE risk was assessed using the Caprini risk assessment model, which incorporates multiple patient- and procedure-related factors to generate a cumulative score. On the basis of the total score, patients were categorized into three risk classes: low (≤2), intermediate (3–4) and high (≥5). For analysis, patients were dichotomized into VTE score ≥ 5 and <5 [20].

DVT was diagnosed on the basis of the results of lower-limb ultrasonography the day before discharge. According to the European Guidelines for the Prevention of Perioperative Venous Thromboembolism, an operation duration ≥ 4 h is a risk factor for VTE in neurosurgical patients [21]. Moreover, a prolonged length of stay (≥12 days) has been shown to be an independent predictor of VTE [22]. Regarding the ultrasonography surveillance protocol, all patients in this study were scheduled to undergo two routine lower extremity venous duplex ultrasound examinations during their hospitalization. The first ultrasonography was performed preoperatively to screen for preexisting DVT. Patients with positive preoperative findings were excluded from the final analysis to ensure that all incident DVT cases in this cohort were new-onset postoperative DVT. A second ultrasonography was performed on the day before discharge to detect postoperative DVT. This two-examination protocol was instituted as a hospital-wide standard for neurosurgical patients at high risk of bleeding who were receiving mechanical prophylaxis, with the aim of systematically capturing both pre-existing and newly developed DVT events.

The major underlying comorbidities recorded were hypertension, diabetes and cardiovascular disease (including ischaemic heart disease, congestive heart failure and a history of stroke or transient ischaemic attack), all of which were defined according to standard diagnostic criteria. The number of underlying diseases, rather than individual conditions, was used as an analytical variable because the frequency of each specific comorbidity was relatively limited for separate multivariable adjustments and the cumulative comorbidity count served as a practical and validated summary measure of the overall disease burden.

### 2.3. Statistical analysis

Baseline sample characteristics were summarized using descriptive statistics and groups were compared using the chi-square test. For multivariable analysis, a single multivariable logistic regression model with DVT as the dependent variable was performed. The primary independent variable was the prophylactic intervention. Odds ratios (OR) with confidence intervals (CI) were used to describe the relationships between covariates and outcomes. Since the main comparison was the odds ratio of DVT for NMES versus IPC, confounding factors could be chosen because they may influence both treatment allocation and DVT and not only because they were associated with the outcome. Because the number of events was limited, a parsimonious model with clinically justified covariates was used. In addition, model calibration was assessed using the Hosmer–Lemeshow goodness-of-fit test, with a *p* value >.05, indicating acceptable model fit. Model performance was further evaluated using the omnibus test, Nagelkerke *R*^2^ test and C-statistic (area under the ROC curve). Sensitivity analysis was performed using a multivariable model to assess the robustness of the findings. All reported *p* values were two-sided, and *p* < .05 was considered to indicate statistical significance. All statistical analyses were performed using SPSS version 29.0.

This study was conducted and reported in accordance with the Strengthening the Reporting of Observational Studies in Epidemiology (STROBE) reporting guideline for cohort studies [23]. The completed STROBE checklist is provided as Supplementary Material. In addition, the GRADE (Grading of Recommendations, Assessment, Development and Evaluation) working group classification was used to assess the quality of evidence for DVT. Study design dictates the baseline quality of evidence; however, other factors can decrease or increase the quality of evidence [24]. For example, unexplained heterogeneity or a high probability of publication bias could decrease the quality of the evidence and a large magnitude of the effect or dose-response gradient could increase it.

## 3. Results

A total of 232 participants were enrolled in this study: 131 patients received IPC and 101 received NMES, with no overlap between the two groups. The mean age of the participants was 62.40 years, and 59.91% were men. At baseline, there were differences in the number of underlying diseases, operation duration, length of stay and disease category between the NMES and IPC groups; participants in the NMES group had fewer underlying diseases, shorter lengths of stay and shorter operation durations, but there were no differences in sex, age, VTE risk score, or type of surgery (Table 1). Of the 4093 patients initially screened, 3861 were excluded according to the criteria described above. Among these excluded patients, a considerable number did not receive either NMES or IPC and were therefore not considered in this comparative analysis. Owing to the retrospective nature of this study and the specific focus on NMES-IPC comparison, detailed baseline clinical characteristics for the entire excluded population were not systematically extracted. Nevertheless, the substantial reduction from 4093 to 232 reflects the highly selective nature of this cohort, which was limited to patients who received one of the two mechanical interventions. These baseline imbalances suggest that the NMES group may represent a clinically milder subgroup, which could partially contribute to the lower DVT rates observed. To account for these differences, all imbalanced baseline variables were included as covariates in the multivariable logistic regression model.

**Table 1.** Baseline characteristics of neuromuscular electrical stimulation (NMES) and intermittent pneumatic compression (IPC) (*n* = 232) patients.

Variables	Total (*n* = 232)	IPC (*n* = 131)	NMES (*n* = 101)	χ^2^	*p*
	*n* (%)	*n* (%)	*n* (%)		
Sex				1.470	.225
Male	139 (59.91)	74 (53.24)	65 (46.76)		
Female	93 (40.09)	57 (61.29)	36 (38.71)		
Age (year)				2.901	.089
<60	89 (38.36)	44 (49.44)	45 (50.56)		
≥60	143 (61.64)	87 (60.84)	56 (39.16)		
Number of underlying diseases				4.575	.032
<2	201 (86.64)	108 (53.73)	93 (46.27)		
≥2	31 (13.36)	23 (74.19)	8 (25.81)		
Venous thromboembolism risk score				0.088	.767
<5	62 (26.72)	36 (58.06)	26 (41.94)		
≥5	170 (73.28)	95 (55.88)	75 (44.12)		
Operation duration (h)				8.674	.003
<4	173 (74.57)	88 (50.87)	85 (49.13)		
≥4	59 (25.43)	43 (72.88)	16 (27.12)		
Length of stay (day)				12.446	<.001
<12	30 (12.93)	8 (26.67)	22 (73.33)		
≥12	202 (87.07)	123 (60.89)	79 (29.11)		
Disease category				6.988	.030
Traumatic brain injury	75 (32.33)	48 (64.00)	27 (36.00)		
Haemorrhagic cerebrovascular disease	127 (54.74)	62 (48.82)	65 (51.18)		
Ischaemic cerebrovascular disease and others	30 (12.93)	21 (70.00)	9 (30.00)		
Type of surgery				4.653	.199
Haemorrhagic cerebrovascular disease surgery	144 (62.07)	75 (52.08)	69 (47.92)		
Ischaemic cerebrovascular disease and tumour/functional surgery	21 (9.05)	15 (71.43)	6 (28.57)		
Traumatic and decompressive/drainage surgery	31 (13.36)	21 (67.74)	10 (32.26)		
No surgery	36 (15.52)	20 (55.56)	16 (44.44)		

As shown in Table 2, incident DVT during the postoperative hospitalization period was observed in 62 of 232 neurosurgical inpatients; of these, 11 received NMES and 51 received IPC. DVT was significantly associated with age [≥60 years (34.97%) vs. <60 years (13.48%)], sex [male (19.42%) vs. female (37.63%)], operation duration [≥4 h (40.68%) vs. <4 h (21.97%)] and length of stay [≥12 d (29.21%) vs. <12 d (10.00%)]. The crude DVT incidence was 10.89% (11/101) in the NMES group and 38.93% (51/131) in the IPC group (*p* < .001).

**Table 2.** Factors related to deep venous thrombosis (DVT) (*n* = 232).

	Total(*n* = 232)	DVT (*n* = 62)	Non-DVT (*n* = 170)	χ ^2^	*p*
Variables	*n* (%)	*n* (%)	*n* (%)		
Sex				9.435	.002
Male	139 (59.91)	27 (19.42)	112 (80.58)		
Female	93 (40.09)	35 (37.63)	58 (62.37)		
Age (year)				12.928	<.001
<60	89 (38.36)	12 (13.48)	77 (86.52)		
≥60	143 (61.64)	50 (34.97)	93 (65.03)		
Number of underlying diseases				1.402	.236
<2	201 (86.64)	51 (25.37)	150 (74.63)		
≥2	31 (13.36)	11 (35.48)	20 (64.52)		
Venous thromboembolism risk score				1.432	.231
<5	62 (26.72)	13 (20.97)	49 (79.03)		
≥5	170 (73.28)	49 (28.82)	121 (71.18)		
Operation duration (h)				7.867	.005
<4	173 (74.57)	38 (21.97)	135 (78.03)		
≥4	59 (25.43)	24 (40.68)	35 (59.32)		
Length of stay (day)				4.921	.027
<12	30 (12.93)	3 (10.00)	27 (90.00)		
≥12	202 (87.07)	59 (29.21)	143 (70.79)		
Intervention				22.191	<.001
IPC	131 (56.47)	51 (38.93)	80 (61.07)		
NMES	101 (43.53)	11 (10.89)	90 (89.11)		
Disease category				0.239	.888
Traumatic brain injury	75 (32.33)	21 (28.00)	54 (72.00)		
Haemorrhagic cerebrovascular disease	127 (54.74)	34 (26.77)	93 (73.23)		
Ischaemic cerebrovascular disease and others	30 (12.93)	7 (23.33)	23 (76.67)		
Type of surgery				2.205	.531
Haemorrhagic cerebrovascular disease surgery	144 (62.07)	41 (28.47)	103 (71.53)		
Ischaemic cerebrovascular disease and tumour/functional surgery	21 (9.05)	6 (28.57)	15 (71.43)		
Traumatic and decompressive/drainage surgery	31 (13.36)	9 (29.03)	22 (70.97)		
No surgery	36 (15.52)	6 (16.67)	30 (83.33)		

*Note:* IPC: intermittent pneumatic compression; NMES: neuromuscular electrical stimulation.

Rather than using these univariable results as the sole basis for variable selection, these covariates were included in the multivariable logistic regression model on the basis of their clinical relevance and baseline imbalance between treatment groups. Table 3 indicates that after controlling for sex, age, operation duration and length of stay, NMES use was associated with lower odds of DVT compared with IPC (adjusted OR = 0.268, 95% CI: 0.125–0.576, *p* = .001). Similarly, male sex was associated with lower odds of DVT (adjusted OR = 0.469, 95% CI: 0.242–0.910, *p* = .025). Older age (adjusted OR = 3.419, 95% CI: 1.594–7.334, *p* = .002) and longer operation duration (adjusted OR = 2.180, 95% CI: 1.042–4.560, *p* = .039) were associated with increased odds of DVT.

**Table 3.** Univariable and multivariable logistic regression analyses of factors associated with deep vein thrombosis (DVT) (*n* = 232).

	DVT [yes (*n* = 62) vs. no (*n* = 170)]			
	Multivariable		Univariable	
Variables	OR (95% CI)	*p*	OR (95% CI)	*p*
Age (≥60 year vs. <60 year)	3.419 (1.594–7.334)	0.002	3.450 (1.715–6.938)	<.001
Sex (male vs. female)	0.469 (0.242–0.910)	0.025	0.399 (0.221–0.723)	.002
Number of underlying diseases (≥2 vs. <2)	–	–	1.618 (0.726–3.605)	.236
Venous thromboembolism risk score (≥5 vs. <5)	–	–	1.526 (0.761–3.061)	.231
Operation duration (≥4 h vs. <4 h)	2.180 (1.042–4.560)	0.039	2.436 (1.295–4.583)	.005
Length of stay (≥12 day vs. <12 day)	3.321 (0.859–12.834)	0.082	3.713 (1.085–12.713)	.027
Intervention (NMES vs. IPC)	0.268 (0.125–0.576)	0.001	0.192 (0.094–0.393)	<.001

*Notes: p* Value for Hosmer–Lemeshow test of multivariable logistic regression = 0.491, Omnibus test: χ ^2^=49.238, *p* < .001, Nagelkerke *R*^2^=0.278, C‑statistic = 0.788. IPC: intermittent pneumatic compression; NMES: neuromuscular electrical stimulation.

The sensitivity analysis in Table 4 further shows that excluding length of stay from the model did not substantially alter the results. NMES remained significantly associated with decreased odds of DVT (OR = 0.230, 95% CI: 0.108–0.488, *p* < .001) and operation duration maintained a borderline significant trend (OR = 2.056, 95% CI: 0.995–4.249, *p* = .051), confirming the robustness of the primary findings.

**Table 4.** Sensitivity analysis: multivariable logistic regression model excluding length of stay (*n* = 232).

	DVT [yes (*n* = 62) vs. no (*n* = 170)]	
Variables	OR (95% CI)	*p*
Age (≥60 year vs. <60 year)	3.315 (1.556–7.065)	.002
Sex (male vs. female)	0.500 (0.260–0.961)	.037
Operation duration (≥4 h vs. <4 h)	2.056 (0.995–4.249)	.051
Intervention (NMES vs. IPC)	0.230 (0.108–0.488)	<.001

*Notes: p* Value for Hosmer–Lemeshow test = 0.508, Omnibus test: χ ^2^=45.615, *p* < .001, Nagelkerke *R*²=0.260, C‑statistic = 0.778. IPC: intermittent pneumatic compression**;** NMES: neuromuscular electrical stimulation.

Table 5 indicates that, on the basis of the GRADE framework, the overall certainty of this body of evidence was rated very low owing to the observational design and substantial methodological limitations.

**Table 5.** GRADE classification of quality of evidence.

Outcome	Author	Year	No. of studies	RCTs	Cohort	Case control	Risk of bias	Inconsistency	Indirectness	Imprecision	Publication bias	Plausible confounding	Magnitude of effect	Dose- response gradient	Quality
DVT	Li et al.	2026	1	0	1	0	Serious risk	Serious inconsistency	No serious indirectness	Serious risk	No	Would not reduce effect	No	No	Very low

## 4. Discussion

The present observational findings support the a priori hypothesis. Among neurosurgical patients with a high bleeding risk for whom pharmacological thromboprophylaxis was not feasible, mechanical prophylaxis with NMES was associated with significantly lower odds of postoperative DVT than with IPC. Beyond this primary observation, male sex, younger age and shorter operation duration were also identified as factors associated with decreased DVT risk within this study sample.

### 4.1. Clinical implications

In the present study, the overall DVT rate was high, particularly in the IPC group. A possible reason is that the majority of these DVTs were asymptomatic and confined to the distal veins, as identified through routine surveillance ultrasonography, rather than triggered by clinical symptoms. This high detection rate is consistent with previous reports indicating that systematic screening of neurosurgical populations yields DVT incidences ranging from 20%–40%, with distal asymptomatic events constituting the majority of cases [6,25]. In addition, the present findings imply that NMES may be a preferable alternative to IPC in this selected population. However, they are exploratory and do not constitute a definitive clinical recommendation. These findings are specific to patients with a high risk of bleeding and should not be applied to all neurosurgical patients receiving standard chemoprophylaxis.

DVT can result from three causal factors: venous stasis, vessel damage and hypercoagulability [26]. The basic mechanism of DVT prophylaxis by IPC is to boost venous flow and velocity [27]. Nevertheless, the external stress exerted by the IPC struggles to affect the deep vein and thus cannot properly prevent DVT in patients [13]. Unlike IPC, NMES can provide substantial augmentation of superficial and deep vein flow and velocity to prevent DVT by delivering specific electrical stimulation to the common peroneal nerve of the lower limbs [14,28]. Additionally, studies have shown that NMES performs better than IPC in terms of boosting the maximum venous flow rate and mean time maximum flow rate [29,30]. Moreover, the fact that NMES stimulates the patient’s muscles rather than passively compressing them is another advantage of NMES over IPC. By stimulating the peroneal nerve, NMES causes the leg muscles to contract regularly, resulting in foot dorsiflexion activity, which promotes vascular expansion to excrete blood in the deep venous system and improves venous flow [31]. Finally, stimulation of the peroneal nerve decreases the level of plasminogen activator inhibitor 1 to enhance thrombolysis [32].

Sex is an important risk factor for DVT [33]. This study showed that women were more prone to DVT than men, which contradicts the results of previous studies. In a review of the risk factors for VTE at the patient level, multiple studies have shown that men have a greater risk of first unprovoked VTE than women [33]. However, a previous study showed that the incidence of DVT in men did not change with age and that the incidence of DVT increased when women were > 60 years old [34]. In this study, the participants were relatively older (mean age 62.40 years), which may be the main reason why women had a greater incidence of DVT than men.

In addition, age is considered an important risk factor for DVT [35] and the primary factor in the VTE risk score. In this study, patients ≥ 60 years of age were at significantly greater risk than younger patients. Previous studies have supported the association between increasing age and greater risk of VTE [36,37]. Age is a risk factor for DVT in neurosurgical patients after surgery [25]. With an increase in the incidence of underlying diseases and a decrease in physical activity, the incidence of DVT in patients in this age group has also increased [38]. Although age is an important risk factor, early intervention in older patients may reduce the incidence of DVT [35].

In neurosurgical patients, operation duration is also considered a significant risk factor for DVT [21]. In this study, it was found that patients who underwent surgery for ≥ 4 h had a greater risk of DVT. A previous study found an association between longer operation duration and a higher 30-day incidence of VTE [39]. Another study reported that the risk of VTE increases when the operation duration is ≥ 310 min [40]. Patients who undergo neurosurgical procedures and have longer than average operation duration are at an increased risk of VTE within 30 days of discharge, even after considering other variables that could affect the outcome [39]. Although the specific threshold varies across studies, the risk of DVT increases with longer operation duration. Prolonged surgery may lead to prolonged immobility, increasing the risk of venous stasis, activation of the coagulation system and damage to the endothelial cells [41]. Additionally, prolonged surgery can trigger an inflammatory response and oxidative stress, which independently promote thrombus formation [42].

From a clinical viewpoint, previous studies have primarily focused on the effectiveness of NMES in orthopaedics [17,43,44], with few studies in neurosurgery. There is a lack of direct comparative evidence between NMES and IPC for DVT prophylaxis. This study provides comparative evidence that may inform the selection of NMES and IPC in this specific clinical context.

### 4.2. Limitations

This study has several limitations. First, the retrospective, non-randomized design predisposed patients to confounding by indication, as patients who received NMES had fewer comorbidities and shorter operations and residual confounding from unmeasured variables could not be excluded [45–47]. Moreover, while the use of NMES was associated with a lower incidence of DVT, the causal relationship remains unclear [48]. Second, the study population is highly selected. Only 232 of the 4093 neurosurgical admissions were included in the study. Patients were excluded if they did not receive NMES or IPC, did not undergo two lower-limb ultrasound examinations, had unavailable or non-diagnostic ultrasound data, or received prophylactic anticoagulation. This creates the risk of selection bias. The third constraint in data collection precluded a thorough analysis of the effects of variables such as NMES intensity and waveform on the effectiveness of DVT prevention. Fourth, 62 DVT events and five predictors were identified. Although the model may not be overfitted, the wide confidence interval for length of stay suggests lay bias estimations. This should be considered when interpreting these findings, as larger studies are required to confirm the stability of these estimates. Fifth, comprehensive model performance evaluations beyond the Hosmer–Lemeshow test were not performed. The low Nagelkerke *R*^2^ in this model suggests that additional unmeasured variables (e.g. emergency/elective procedure, malignancy, neurological deficit, immobility, ICU stay, prior VTE, BMI and contraindications to anticoagulation) may need to be considered in future studies to improve explanatory power. Finally, owing to the retrospective nature of the data collection, information on DVT-related symptoms may not have been consistently documented in all patients’ medical records. Therefore, it was not only difficult to reliably classify DVTs as symptomatic or asymptomatic but also to reliably retrieve detailed information on the device type, timing and duration of NMES and IPC applications in this study. Further prospective studies should adopt standardized protocols with detailed documentation of intervention parameters.

### 4.3. Conclusion

In conclusion, this study found an association between NMES and reduced odds of DVT compared with IPC in patients undergoing neurosurgery. Although NMES may be more widely adopted in neurosurgery to decrease the incidence of DVT, further evidence is required to determine its effectiveness in clinical practice.

## Supplementary Material

STROBE_checklist_cohort.docx

## Data Availability

The data that support the findings of this study are not publicly available because they contain information that could compromise the privacy of the research participants but are available from the corresponding author upon reasonable request.
